# Assessment of Zataria Multiflora Essential Oil—Incorporated Electrospun Polyvinyl Alcohol Fiber Mat as Active Packaging

**DOI:** 10.3390/polym15041048

**Published:** 2023-02-20

**Authors:** Farid Moradinezhad, Sara Hedayati, Elham Ansarifar

**Affiliations:** 1 Department of Horticultural Science, Faculty of Agriculture, University of Birjand, Birjand 9717434765, Iran; 2 Nutrition Research Center, Shiraz University of Medical Sciences, Shiraz 7134814336, Iran; 3 Department of Food Hygiene and Quality Control, School of Nutrition and Food Sciences, Shiraz University of Medical Sciences, Shiraz 7134814336, Iran; 4 Social Determinants of Health Research Center, Department of Public Health, School of Health, Birjand University of Medical Sciences, Birjand 9717853076, Iran

**Keywords:** polyvinyl alcohol, Zataria multiflora essential oil, electrospinning, fruit preservation, strawberry

## Abstract

In this study, an active packaging containing Zataria multiflora essential oil (ZMEO), a powerful natural antimicrobial agent, encapsulated into polyvinyl alcohol (PVA) fiber via electrospinning is presented. ZMEO was effective on pathogenic bacteria, particularly Gram-positive bacteria (*Staphylococcus aureus*, *Bacillus cereus*, *Listeria monosytogene*), fungi and yeasts (*Aspergillus fumigatus, Candida albicans*). Results showed that the scanning electron microscopy (SEM) images of fibers had a bead-free and uniform structure. Fourier-transform infrared (FTIR) revealed that ZMEO was encapsulated into PVA through a physical process, without chemical interaction between the ingredients. Strawberries treated with PVA/ZMEO significantly (*p* < 0.05) preserved the anthocyanin (18.64%), total phenols (12.95%), antioxidant (22.72%), soluble solids (6.44%), titratable acidity (20.88%), firmness (27.2%), and color (15.55%) compared to the control sample during 15 days of cold storage. According to these findings, electrospinning was an efficient method for encapsulating bioactive compounds. ZMEO loaded into PVA fiber delayed the physiological and biochemical changes of fruits and extended the fruit’s shelf-life. This study revealed the benefits of incorporating ZMEO into PVA fiber mats, which could lead to new possibilities for active packaging.

## 1. Introduction

The strawberry (*Fragaria ananassa*) is a nutrient-rich and highly perishable fruit [1,2]. The rise in global demand for fresh fruits and vegetables during the last four decades has resulted in the use of different preservation technologies: active packaging [3], controlled atmospheres [4], and edible coating [5,6].

Packaging is used to preserve foods from environmental impacts and microbial contamination in order to protect the quality and safety of food products, to avoid their spoilage and to increase their shelf-life [7]. Bioactive packaging is developing to additionally provide antibacterial and antioxidant activity with the same goals, i.e., extending the shelf-life while ensuring the safety of the food products [7,8]. Aromatic oily liquids, called essential oils (EOs), are obtained from different plant parts. Previous studies have demonstrated that EOs have antioxidant, antibacterial, antiparasitic, insecticidal, and antifungal properties [9,10]. *Zataria multiflora Boiss* (avishan-e shirazi or zaatar) belongs to the family Lamiaceae, and it is a native Iranian plant with a strong aroma [11]. Ardekani et al., (2018), Lin et al., (2018), Vafania et al., (2019) and Ansarifar et al., (2022) studied the application of the nanoencapsulation of Zataria multiflora essential oil in electrospun polyvinyl alcohol as a potential wound dressing [12], in gelatin fibers as active packaging against *Campylobacter jejuni* in chicken [13], in chitosan–gelatin fibers in order to reduce nitrite in sausages [14], and in electrospun polyvinyl alcohol as the active packaging and preservation of strawberry fruits, respectively [15].

Encapsulation improves the stability of the essential oil against environmental degradation and masks some of the undesirable flavor as well [16]. Among various approaches for encapsulation, electrospinning is a simple and effective method for preparing polymer fibers and the encapsulation of bioactive components [1,17,18]. Many reports have used electrospun fibers for fruit preservation; for example, Shao et al., (2018) used tea polyphenols’ encapsulation into pullulan–CMC electrospun fiber [19], Zhang et al., (2019) encapsulated thymol in fiber via coaxial electrospinning [10], Wang and Lu (2018) showed tea polyphenol-loaded porous core–shell structured fibers were successfully prepared via coaxial electrospinning [20], and Niu et al., (2020) studied electrosprayed particles as encapsulation systems of bioactives [21]. Zeinali et al., (2021) reported the possibility of fabrication and application of PVA nanofiber-containing jujube extract (JE) and the PVA/JE nanofiber displayed a significant effect on extending the shelf-life and preserving the quality of strawberries during storage time [22].

Polyvinyl alcohol (PVA) is a safe and FDA-approved polymer with high water solubility, chemical resistance, and low environmental impact, which is commonly used in the food packaging industry and in medical devices [1,23]. Electrospun PVA fibers have been widely used due to their biodegradability and biocompatibility [1,24]. Chi et al., (2022) fabricated a new hydrogel nanofiber from gelatin and polyvinyl alcohol via electrospinning [18]. Amorim et al., (2022) produced composites from oxidized-bacterial cellulose (BC) and poly (vinyl alcohol)–chitosan (PVA-CH) nanofibers via needleless electrospinning and functionalized with the bacterial pigment prodigiosin (PG). The fabricated composites can be considered for application in active food packaging, owing to PG antimicrobial potential, to prevent foodborne pathogens, but also to prevent external contamination by tackling the exterior of food packaging materials [25].

A recent review reported that combinations of electrospun fibers and EOs had a positive impact on the prolongation of shelf-life and maintaining the quality of food products when used in food packaging [26]. However, there is little information regarding the application of ZMEO for preparing electrospun antimicrobial packaging material. Moreover, there is no report on its effect on strawberries packed with PVA/ZMEO fiber film. Therefore, this research aimed to evaluate the potential application of a novel antimicrobial PVA/ZMEO fiber film fabricated via electrospinning for extending the shelf-life and preserving the quality of strawberries.

## 2. Materials and Methods

### 2.1. Solution Preparation

In order to prepare a PVA/ZMEO solution, PVA (polyvinyl alcohol, Mw = 130,000 Da, Aldrich Chemical CO, St. Louis, MO, USA) (10 g) was dissolved in 100 mL deionized water at 80 °C; then, it was magnetically stirred for 2h until production of homogeneous solution. Zatara multiflora essential oil (ZMEO) containing thymol (54.11%), carvacrol (14.06%), p-cymene (12.1%), linalool (7.43%), and y-terpinene (3.34%) with other minor compounds was obtained from Barij. Co. ZMEO (4 % *w*/*v*) was added to PVA solution at 25 °C and mixed for 24 h.

### 2.2. Electrospinning Process

A 10mL syringe equipped with a stainless-steel needle was used for the electrospinning of the PVA solution with and without ZMEO. In order to produce fibers, electrospinning with a high voltage (model ES-1000, Nanoscale technologists CO, Tehran, Iran) was applied. The applied voltage, rate injection, and distance between the needle tip and the collector were set at 15 kV, 0.5 mL/h, and 15 cm, respectively. Positive and negative electrodes of high voltage were connected to the capillary, and a rotating collector was wrapped with aluminum foil to collect fibers, respectively. The electrospinning experiments were performed at 24 °C and 21% relative humidity.

### 2.3. Antimicrobial Activity of ZMEO

The antimicrobial activity of ZMEO was studied using the following bacteria: *Staphylococcus aureus* (ATCC 29213), *Bacillus cereus* (PTCC 1154), *Listeria monosytogene* (ATCC 35152), *Pseudomonas aeruginosa* (ATCC 13525), *Escherichia coli* (ATCC 11775), *Aspergillus fumigatus* (ATCC 1022), and *Candida albicans* (ATCC 10239). In order to determine the minimum inhibitory concentration (MIC), a broth microdilution assay was employed [14].

### 2.4. Characterization of Fibers

The morphology of electrospun PVA and PVA/ZMEO fibers was examined using a scanning electron microscope (Leo 1450VPSEM) at an acceleration voltage of 20 kV [24]. Fourier-transform infrared FTIR spectra of powder PVA, ZMEO, and PVA/ZMEO fiber were obtained using a Fourier-transform spectrophotometer (Shimadzu 6650, Kyoto, Japan), at 4 cm^−1^ resolution in the frequency range of 400 and 4000 cm^−1^ [23]. The thermal stability of PVA, ZMEO and PVA/ZMEO fibers were evaluated using differential scanning calorimetry (NETZSCH STA 449F3, Selb, Germany). A sample (about 5 mg) was heated from 20 to 400 °C at a heating rate of 10 °C/min under a constant flow rate of nitrogen gas of 20 mL/min [23]. The release profile of ZMEO from PVA fiber mat was studied in 10% (*v*/*v*) aqueous ethanol solution [19]. A total of 20 mg of the PVA/ZMEO fiber was placed in a glass vial containing 20 mL of ethanol (10%) and stirred at 120 rpm. An aliquot of the fiber was extracted every 2 h and absorbance was measured using UV spectroscopy at 278 nm for 180 h [10].

### 2.5. Preparation and Evaluation of Strawberry

Strawberries were selected based on uniformity in ripeness, size, appearance, and without fungal spoilage. After immersing fruits in a solution of 0.1% sodium hypochlorite for 1 min, they were divided into three groups (seven strawberries per group for each replicate) and placed in polyethylene containers (dimension of 11.5 × 9.5 × 6.2 cm). Group control: the fruit packed in PET container without a film on its lid; group PVA: the fruit packed in PET container with a PVA fiber film on its lid; and group PVA/ZMEO: the fruit packed in PET container with ZMEO loaded into a PVA fiber film on its lid. Packages were stored in a cold room at 4 ± 0.5 °C and 85% RH for 15 days. Physicochemical properties of fruits were measured at different time intervals on days 0, 3, 6, 9, 12 and 15, while microbial load of the treated fruit and control was evaluated on days 3, 9 and 15 of storage.

The weight loss of strawberries was calculated using a digital balance (UWA-K-015, China) at each sampling day [5]. Samples were crushed and their juice was collected. Total soluble solids (TSS) of the juice were determined using a hand-held refractometer (Atago, Tokyo, Japan) at 20 °C. Titratable acidity (TA) was assessed by titrating the diluted juice (5 to 95) up to pH 8.2 using 0.1 N NaOH [9]. The anthocyanin content of fruits was determined using the pH differential method described by the authors of [27]. The total phenol content of fruits was measured using the Folin–Ciocalteu method [28]. The antioxidant capacities of the fruit samples were determined using 2,2-diphenyl-1-picrylhydrazyl (DPPH) radical scavenging assay [29]. The firmness of fruits was calculated using a digital penetrometer (FHT200, Extech Co., USA), fitted with a cylinder probe with a diameter of 2 mm. The surface color parameters of fruits were measured with a colorimeter (TES 135-A, Taiwan) [5]. Microbial analysis of strawberries was performed using the colony counting method: Total bacteria and total fungi and yeast were measured on the plate count agar (Merck 1.05463 PCA) incubated at 37 °C for 2 days, and on potato dextrose agar (Merck 110130 PDA) incubated at 28 °C for 3 days, respectively. A 5–1 scale (5 = no defects; 4 = minor defects; 3 = moderate defects (limit of marketability); 2 = major defects (limit of edibility); and 1 = inedible) was used to determine the effect of storage and packaging on overall appearance of the strawberries

### 2.6. Statistical Analysis

The data were analyzed using the statistical software SPSS (IBM SPSS Statistics, Version 22, New York, NY, USA). The analysis of variance (one-way ANOVA) and Duncan’s test and post hoc test were used to analyze significant differences between the samples. *p* ˂ 0.05 was considered the significance level.

## 3. Results and Discussion

### 3.1. Antimicrobial Activity of ZMEO

The results of test antimicrobial activity of ZMEO against various Gram-positive and Gram-negative food-borne bacteria are presented in Table 1. Minimal inhibitory concentration (MIC) was studied via agar disc diffusion [14]. As can be seen in Table 1, the MIC was 4.32 ± 0.32, 4.42 ± 0.25, 3.52 ± 0.61, 8.61 ± 0.22, 5.35 ± 0.15, 1.06 ± 0.32 and 0.85 ± 0.25 mg/mL for *Staphylococcus aureus, Bacillus cereus, Listeria monosytogene, Pseudomonas aeruginosa, Escherichia coli, Aspergillus fumigatus,* and *Candida albicans.* According to the results, it was found that the Gram-negative bacteria were the most resistant, the Gram-positive bacteria exhibited moderate sensitivity, and fungi and yeasts were the most susceptible ones. The resistance of Gram-negative bacteria might have resulted from the presence of hydrophilic properties of their impermeable outer membrane to lipophilic compounds such as essential oils [30]. Carvacrol, terpinene, and thymol are the main components of ZMEO that had good antimicrobial agents in previous studies [30,31]. A study reported that *Staphylococcus aureus, Salmonella typhi,* and *Escherichia coli* were more susceptible to ZMEO [31]. Another study showed that *Zataria multiflora* essential oil was effective on pathogenic bacteria, particularly *Staphylococcus aureus* and *E. coli* [30].

### 3.2. Scanning Electron Microscopy (SEM)

**Figure 1:**
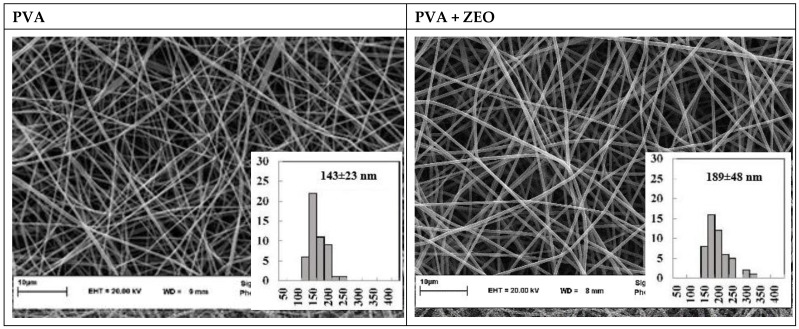
SEM image of PVA and PVA/ZMEO nanofibers, (mean ± SD).

The morphology and diameter of fibers are effective factors in the final properties of electrospinning film [24]. The SEM image and diameter distribution of PVA and PVA/ZEMO fiber are shown in Figure 1; as can be seen, fibers had a smooth surface, linear morphology, and a bead-free structure. The fiber average diameter of PVA and PVA/ZMEO samples (n = 50) was 143 ± 23 and 189 ± 48 nm, respectively. The result demonstrated that the ZMEO was well-loaded into the fibers, and that by adding ZMEO in fiber, the average diameter significantly (*p* ˂ 0.05) increased. This is attributed to the fact that by adding essential oil, the decrease of electrical conductivity (from 0.5 to 0.37 mS/cm) and surface tension (from 48 to 44 mN/m) and increase in viscosity (from 0.1 to 0.13 pa·s) consequently reduced the elongation of polymer jet by the applied voltage; therefore, the diameter of the fiber increased. This result was similar to the reports of Mohammadi et al., (2016) and Ataei et al., (2020) regarding adding essential oil into PVA fiber [11,26].

### 3.3. Fourier-Transform Infrared (FTIR)

**Figure 2:**
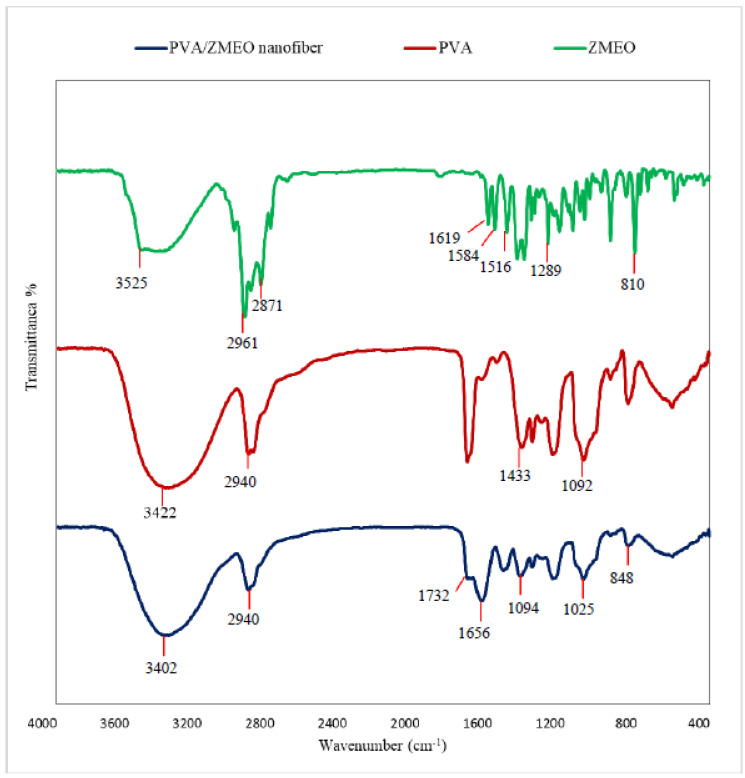
Fourier-transform infrared (FTIR) spectra for PVA powder, ZM essential oil, PVA/ZMEO fiber.

The FTIR spectrum of PVA powder, ZMEO, and PVA/ZMEO fiber is shown in Figure 2. The FTIR spectra of PVA powder had the characteristic peaks at 3422 cm^−1^ (O–H), 2940 cm^−1^ (C–H), 1433 cm^−1^ (CHOH), and 1092 cm^−1^ (C–O) [12,32]. The FTIR spectra of the pure ZMEO showed the peak at 1516 (C=C aromatic ring), 1289 cm^−1^ (aromatic ether), 810 cm^−1^ (CH wagging vibrations), 1584 (N–H bending), 1619 (conjugated double bond of the ring), 2961 cm^−1^, 2871 cm^−1^ (asymmetric and symmetric methyl C–H stretching), and 3525 cm^−1^ (O–H vibration of hydroxyl group) [11,14].

As seen in Figure 2, the spectrum of PVA/ZMEO fiber showed all characteristic peaks of the PVA and ZMEO and no new peak was overserved; this confirms that ZMEO is successfully encapsulated into PVA fiber without chemical modification. Furthermore, the spectrum obtained for the PVA/ZMEO fiber showed a band close to 840 cm^−1^ related to C-H bending of the ring in the ZMEO structure that related to the antimicrobial properties of ZMEO. Ansarifar et al., (2022), Ataei et al., (2020) and Mohammadi et al., (2016) considered encapsulation of essential oil in PVA fibers via electrospinning and reported that the characteristic peaks of PVA and essential oil had no remarkable change after the process, indicating the successful encapsulation via physical interactions [11,15,26]. Moreover, Bose et al., (2019) and Lin et al., (2018) examined encapsulation of thyme essential oil in chitosan–gelatin fiber and cinnamaldehyde in zein fiber. They reported that wall material and essential oils were mixed without any chemical interaction [13,28].

#### Differential Scanning Calorimetry (DSC)

**Figure 3:**
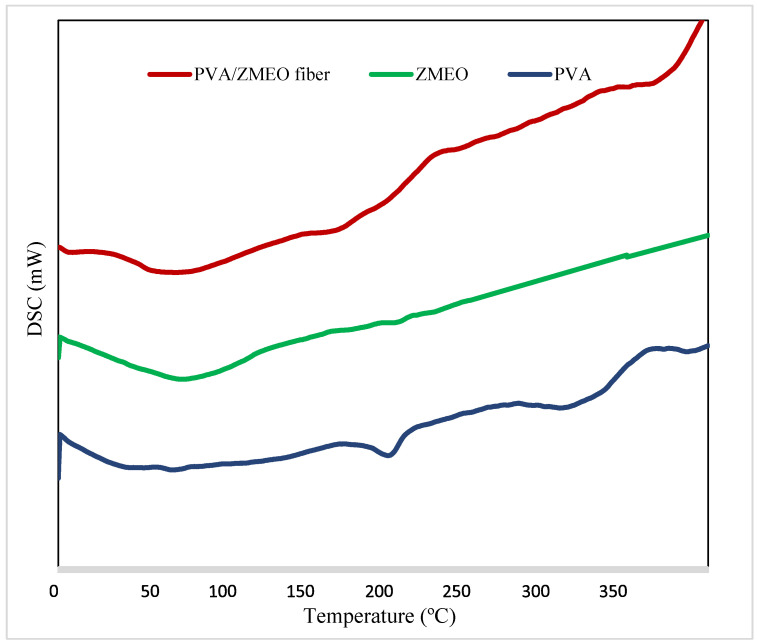
DSC (differential scanning calorimeter) thermograms for PVA powder, ZM essential oil, PVA/ZMEO fiber.

The DSC thermograms of PVA powder, ZM essential oil and PVA/ZMEO nanofiber presented in Figure 3 PVA displayed an endothermic glass transition peak and a sharp endothermic melting transition peak around 57 °C and 196 °C. The first peak is determined to be a thermal effect due to moisture evaporation from the sample and may also be due to a glass transition, and the second peak is endothermic melting transition at 196 °C, which may be related to the total degradation temperature of PVA. The temperature required for melting of 100% crystalline PVA is 306 °C (Table 2). Berna et al., (2019) and Wen et al., (2017) reported similar peaks for the thermogram of PVA.

Examination of the thermogram of the ZMEO shows that this essential oil has an endothermic peak at 95.6 °C. This peak is probably related to the evaporation temperature of the essential oil and its volatile parts, which indicates volatility and thermal instability of the ZMEO. ZMEO depicted one endothermic peak at 88 °C corresponding to its melting point [33].

PVA/ZMEO displayed three endothermic peaks around 61 °C, 246.7 °C and 357.3 °C (Table 2), which represent crystallization and melting points. The starting point of main weight loss of PVA/ZMEO nanofiber was more than ZMEO. This indicated the encapsulation of ZMEO into PVA nanofiber to increase its thermal stability. The results of Charpashlo et al., (2020), Karim et al., (2020) and Berna et al., (2019) on PVA nanofiber–thymol essential oil, zein nanofiber–cinnamic aldehyde and zein–gelatin nanofiber–lycopene were similar to the results of this study [33,34,35].

### 3.4. Release Profile of ZMEO

**Figure 4:**
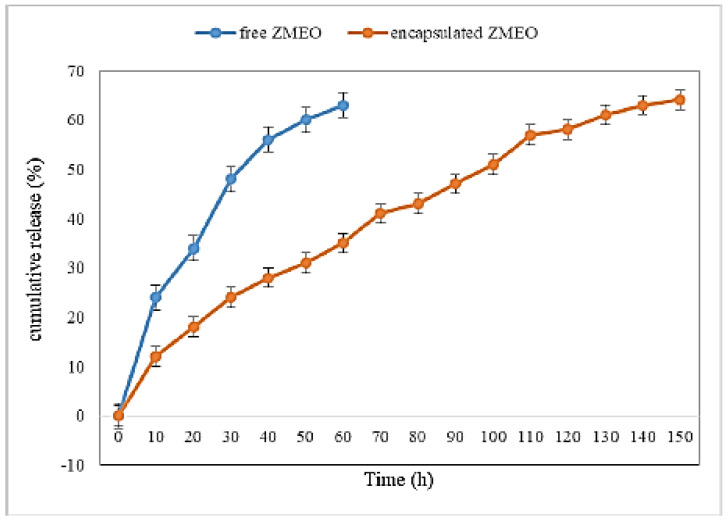
Release profile of ZMEO free and encapsulated into PVA fiber film.

The encapsulation efficiency of ZMEO in the PVA fiber mat was approximately 83.42 ± 21.1%. The release profile of ZMEO in two states (free and encapsulated) was evaluated for 150 h. As shown in Figure 4, free ZMEO was released rapidly at room temperature, with 63% of it having been released after 60 h, while ZMEO encapsulated in PVA fiber mat was released into the atmosphere much more slowly, with only 64% of it having been released after 150 h. ZMEO release was controlled due to the formation of a core–shell structure by the PVA fiber mat during electrospinning [1,10]. Zhang et al., (2019) reported that thymol released quickly within 19 h and thymol encapsulated in fiber was released much more slowly (36% of it during 72 h) [10].

### 3.5. Fruit Physicochemical Measurements

#### 3.5.1. Weight Loss

As expected in all treatments, the fruit weight loss significantly (*p* ≤ 0.05) increased as the storage time increased (Table 3). The fruit stored in the containers with fiber exhibited less weight loss compared to the control. All treatments had significantly lower weight loss compared to the control samples during the storage period. Strawberries packed with PVA fiber showed the lowest weight loss (2.22%) on day 3 of storage (Table 3), while the highest weight loss (14.56%) was observed in the control group on day 15 of storage. Both PVA and PVA/ZMEO treatments reduced the respiration rate of the fruits. In the PVA/ZMEO treatment, ZMEO is released from the fiber slowly, delaying the ripening process of fruits; thus, there was significantly less weight loss from day 6 to the end of storage period. Researchers found that treating strawberry fruits with thyme essential oil significantly decreased the fruit weight loss compared to the control [19]. The interaction between treatments and storage durations was also significant (*p* ≤ 0.05) in terms of weight loss (Table 3). The fruit weight loss gradually increased as the storage time progressed. The fruit weight loss occurred during storage due to its respiratory process. In accordance with Dhital et al.’s (2018) report on strawberries, in the current study, the highest weight loss was observed on the 15th day of storage in all treatments [2]. However, the fruit packed with fiber (PVA or PVA/ZMEO) had significantly less weight loss than the control during the storage period. Generally, the lower weight loss leads to a longer storage life and marketability of fresh fruits.

#### 3.5.2. Firmness

The biggest change occurring in fruits during storage is a loss of firmness. As can be seen in Table 3, all treatments (*p* ≤ 0.05) had significantly higher firmness compared to the control samples from day 3 of storage until the end of the storage period. The fruit treated with fiber showed higher firmness compared to the control during 15 days of cold storage. The highest firmness (1.25 N) was obtained in all treatments after treatment on day 0, and then gradually decreased as the storage time increased. ZMEO released from the fiber maintained the integrity of cell walls of fruit in PVA/ZMEO treatment and decreased the moisture loss. Dong et al., (2017) reported that the firmness of peach fruit declined from 34 to 17 N after 12 days of storage in the control samples, while fruit packed in a container including electrospun fiber-encapsulated hexanal had significantly higher firmness [29]. The firmness of treated fruit remained higher than that of the control during the storage period. However, on day 15, there was no significant difference between the firmness of the packed fruit with PVA and that of the PVA/ZMEO treatment (Table 3). Min et al., (2021) also reported that the softening of strawberry fruit prolonged and the loss of firmness significantly reduced when packaged in thyme essential oil encapsulated in fibers with different composite films [3].

#### 3.5.3. Color Attributes

L*, a* and b* attributes of the fruit skin color were impacted by both active packaging treatments and storage time (Table 3). There was a significant (*p* ≤ 0.05) reduction in L* and b* values with increased storage time for all treatments, while the a* value increased from day 3 of storage and then gradually decreased. These changes showed darker and redder fruit with storage. After 15 days of storage, fruit stored in the container with PVA/ZMEO fiber resulted in a lighter color (higher L* value) than that of the control and PVA treatment with darker color (lower L* value). On day 15, the highest L* (35.92) was observed in the PVA/ZMEO treatment. Similarly, the highest a* value was obtained from day 6 of storage and afterward in the PVA/ZMEO treatment. Saei-Dehkordi et al., (2019) also reported that fig fruits coated with a combination of chitosan and thymol had a significantly higher L* value than the control and individual application of both treatments [30]. These results are in agreement with those of Li et al., (2018), who stated that postharvest treatments of strawberry delayed fruit senescence in which the skin color was lighter than the control fruit [8].

#### 3.5.4. Total Soluble Solids

The results showed that TSS remained steady in all active packaging treatments compared to the control, and there was no significant (*p* > 0.05) difference between all treatments. However, it declined gradually during the storage period (Table 4). The TSS percentage of strawberries significantly decreased after 6 days of storage. This reduction might be due to the sugar metabolism during the respiratory process [9,29,36]. After 15 days of storage, strawberry fruit packed in the container with fiber PVA or PVA/ZMEO had higher TSS compared to the control samples, which is probably due to the slowing down of respiration and metabolic activity in strawberry fruit [24,29].

#### 3.5.5. Titratable Acidity

Since citric acid is the most prevalent organic acid in strawberries, titratable acidity is expressed as the total quantity of citric acid present in the fruit [9]. Titratable acidity was significantly (*p* ≤ 0.05) affected by both storage time and active packaging treatments (Table 4). The initial TA values of fruits (0.72) were the same in all treatments. In all treatments, the TA gradually reduced during storage. Organic acids that act as substrates for the respiratory process during ripening are responsible for this reduction, thus causing the fruit to taste sweeter [37]. After 15 days of storage, fiber PVA or PVA/ZMEO treatments had significantly higher TA compared to the control. This showed that the fruit packed in the container with PVA and PVA/ZMEO significantly maintained the TA compared to control. Moreover, the rate of TA reduction was faster in control samples compared to fruit packed in electrospun fibers of PVA and PVA/ZMEO. The TA of PVA/ZMEO treatment was the highest at the end of the storage period. It is possible that ZMEO delayed the respiratory speed of strawberries during the storage period by reducing the TA consumption [37]. These results indicated that packaging films could extend the strawberries’ shelf-life by delaying the consumption of the nutrients [22,36].

#### 3.5.6. Anthocyanin

The total anthocyanin content of strawberry was impacted by both active packaging treatments and storage time (*p* ≤ 0.05) (Table 4). However, the interaction between treatments and storage duration was not significant (*p* >0.05). The initial anthocyanin content (273.6 mg 100 g FW) was similar in all fruits. After 3 days of storage, the anthocyanin content of all treatments decreased, while the fiber PVA/ZMEO treatment gave significantly higher anthocyanin until the end of the storage time. On day 15, the highest anthocyanin (231 mg 100 g FW) was found in PVA/ZMEO treatment. All fiber treatments significantly preserved the anthocyanin content compared to the control. Saei-Dehkordi et al., (2010) showed that fig fruits coated with a combination of thymol and chitosan exhibited significantly higher anthocyanin content than the control [30]. The anthocyanin content of the fruits significantly decreased during storage. Similar to the results of the current study, Mohammadi et al., (2016) and Hadipour-Goudarzi et al., (2014) indicated that the total anthocyanin content of strawberry fruits decreased gradually during cold storage [11,32]. They stated that low-temperature storage significantly reduced the anthocyanin content in strawberry fruit. Stability of anthocyanin can be affected by other different factors, such as pH, TA, TSS, oxygen, and light [2,9,36]. Increased pH or TSS might adversely affect the stability of anthocyanin [2].

#### 3.5.7. Antioxidant Activity

The antioxidant activity of strawberry was affected by both active packaging treatments and storage time (Table 4). The initial antioxidant capacity (35.21%) was similar, and no statistically significant differences were observed between the packed strawberries in the container with fiber PVA and PVA/ZMEO treatments and control. The antioxidant activity of fruits was improved during the storage time from day 3, and the highest was obtained on day 6; then, it slowly decreased in all treatments until the end of the storage time (Table 4). The highest antioxidant capacity (83.5%) was obtained in PVA/ZMEO treatment. Fiber treatments were significantly more effective at preserving antioxidant capacity than control. Berna et al., (2019) reported that different essential oils such as basil, oregano, and thyme significantly increased the antioxidant capacity in LDPE active packaging [34]. Similarly, Charpashlo et al., (2020) reported that the antioxidant activity of sweet cherries coated with chitosan nanoparticles containing encapsulated *Eryngium campestre* EO moderately increased during 21 days of storage compared to that of the control [35].

#### 3.5.8. Total Phenolic (TP)

As seen in Table 4, there was an increase in the TP of strawberries until the 6th day of storage; then, it gradually reduced in all treatments. This is in agreement with the results of [9], which stated that the TP content of fruits in nano-packaging initially increased and then reduced during cold storage. Considering that the strawberries used for the study had not reached full maturity, TP was most likely gathered initially and resulted in increased levels. In ripened strawberry fruit, TP levels will continue to decline during storage. According to the results (Table 4), the control sample had significantly (*p* ≤ 0.05) lower TP than the active packaging treatment samples. The PVA/ZMEO treatment had the highest TP (1530 mgL^−1^ GA 100 g FW) on day 6 of storage. In other words, fiber mat treatments significantly preserved the TP content of strawberries. In modified atmosphere packaging, oxygen content is reduced, which results in the oxidation of TP content of fruits [2,9]

### 3.6. Microbial Analysis and Overall Appearance

**Figure 5:**
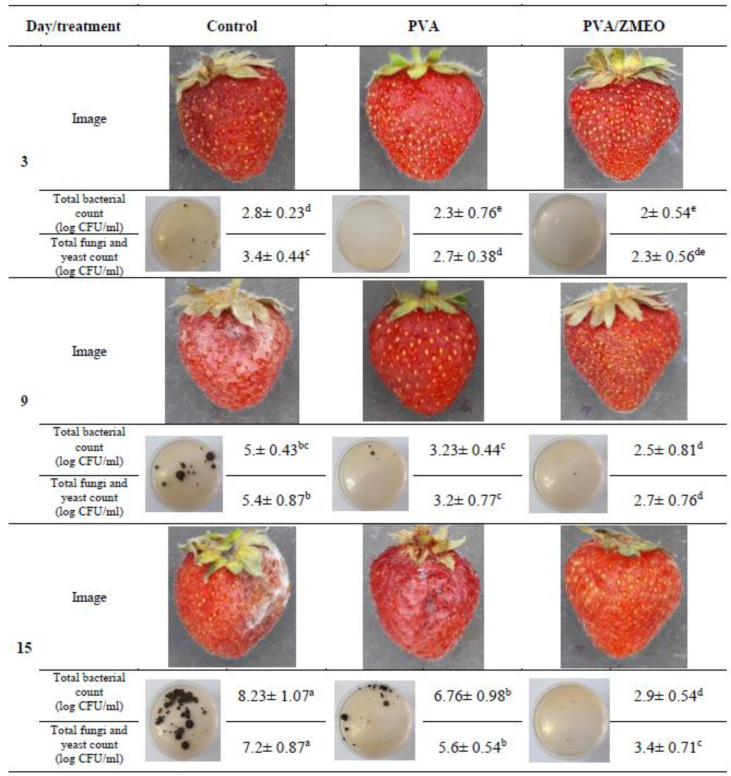
Overall appearance, total bacteria count, total fungi and yeast count of strawberries packed using PVA nanofiber (PVA) and PVA nanofiber loaded with *Zataria Multifora* essential oil (PVA/ZMEO) during 15 days of storage at 4 °C. Data shown are the mean ± standard error of three replicates. ^a–e^ Different letters indicate significant difference for the storage time for each packaging treatment.

Strawberry is an exceptionally perishable fruit with a limited shelf-life after harvest; therefore, it is used to evaluate the efficiency of active packaging [19]. The total microbial load of strawberries was investigated on 3, 9 and 15th days of storage. As shown in Figure 5, the growth rate of the microbial load on strawberries of PVA/ZMEO group was the lowest because the release of ZMEO from fibers inhibit the growth of microorganisms. Moreover, according to Figure 5, strawberries in the control group had decayed, and their appearance was unacceptable on the 9th day of storage; hence, they cannot be consumed. On the other hand, fruit in the PVA and PVA/ZMEO treatment groups was considered suitable for marketing at the end of the storage period. PVA/ZMEO electrospun fiber enhanced the shelf-life of strawberry via two mechanisms: (1) The PVA and PVA/ZMEO fiber mat absorbed moisture from the respiration of the strawberry inside the package. In control packages, there was sufficient moisture for fungi to grow [31]; (2) Carvacrol and thymol are the major components of ZMEO that have antioxidant and antimicrobial properties [12,31]. ZMEO encapsulation in the PVA fiber mat can gradually release into stored fruit packages, slowing down the ripening process of the treated fruit [22]. Thus, ZMEO significantly reduced microbial growth and maintained the firmness and weight of strawberry. In the control group, the rate of respiratory is high. Strawberry quality and overall appearance could be adversely affected by high-moisture conditions because microorganisms grow rapidly. The results agreed with those of Zhang et al., (2019), indicating that essential oil loaded into fiber mat was gradually released during storage; therefore, fruits can be stored for a long period of time [10].

## 4. Conclusions

In this study, we demonstrated that PVA/ZMEO fiber mat can be fabricated using an electrospinning method. The results of the antimicrobial ZMEO found that the Gram-negative bacteria were the most resistant, the Gram-positive bacteria exhibited moderate sensitivity, and fungi and yeasts were the most susceptible ones. The SEM of fiber showed a linear morphology and a bead-free structure. The FTIR and DSC analysis showed that ZMEO was encapsulated into the PVA fiber mat successfully. Free ZMEO was released rapidly at room temperature, with 63% of it having been released after 60 h, while ZMEO encapsulated in PVA fiber mat was released into the atmosphere much more slowly, with only 64% of it having been released after 150 h. ZMEO encapsulated in the PVA fiber mat was slowly released into the package of stored fruit; therefore, it exhibited an antimicrobial effect during long-term storage and had a protective effect on fresh fruits. Strawberries treated with PVA/ZMEO significantly (*p* < 0.05) preserved the anthocyanin (18.64%), total phenols (12.95%), antioxidant (22.72%), soluble solids (6.44%), titratable acidity (20.88%), firmness (27.2%), and color (15.55%) compared to the control sample during 15 days of cold storage. The novel active packaging with excellent release properties can manage the active component and could be used in a wide range of fruit-preservation applications.

## Figures and Tables

**Table 1:** Antimicrobial activity (MIC) of ZMEO.

Microbe	MIC (mg/mL)
*Staphylococcus aureus*	4.32 ± 0.32
*Bacillus cereus*	4.42 ± 0.25
*Listeria monosytogene*	3.52 ± 0.61
*Pseudomonas aeruginosa*	8.61 ± 0.22
*Escherichia coli*	5.35 ± 0.15
*Aspergillus fumigatus*	1.06 ± 0.32
*Candida albicans*	0.85 ± 0.25

Data shown are the mean ± standard error of three replicates.

**Table 2:** Parameters of DSC thermogram for PVA powder, ZM essential oil, PVA/ZMEO fiber.

	T_eo_ (°C)	T_g_ (°C)	H (J/g)	T_eo_ (°C)	T_g_ (°C)	H (J/g)	T_eo_ (°C)	T_g_ (°C)	H (J/g)
PVA	48.3	57.7	1.6889	185.2	196.0	9.847	29.4	306	5.933
ZM essential oil	77.5	95.6	7.352						
PVA/ZMEO fiber	35.0	61.0	26.880	247.1	246.7	5.955	32.6	357.3	3.866

**Table 3:** Effect of active packaging using PVA fiber (PVA) and PVA fiber loaded with *Zataria Multifora* essential oil (PVA/ZMEO), and storage time on weight loss, firmness and color parameters of strawberry fruits stored at 4 °C for 15 days.

Parameter	Active Packaging Treatment	Storage Time (Day)					
		0	3	6	9	12	15
Weight loss (g)	Control	-	3.66 ± 0.34 ^eA^	9.54 ± 0.01 ^dA^	14.05 ± 0.04 ^bA^	13.67 ± 0.31 ^cA^	14.56 ± 0.18 ^aA^
	PVA	-	2.22 ± 0.01 ^dC^	8.27 ± 0.09 ^cB^	11.83 ± 0.08 ^aB^	11.03 ± 0.00 ^bB^	12.03 ± 0.00 ^aB^
	PVA/ZMEO	-	2.94 ± 0.06 ^eB^	6.66 ± 0.15 ^dC^	10.16 ± 0.15 ^cC^	10.48 ± 0.13 ^bC^	11.08 ± 0.00 ^aC^
Firmness (N)	Control	1.25 ± 0.00 ^aA^	0.56 ± 0.04 ^bB^	0.49 ± 0.23 ^bC^	0.49 ± 0.02 ^bC^	0.33 ± 0.01 ^cC^	0.36 ± 0.05 ^cB^
	PVA	1.25 ± 0.00 ^aA^	0.73 ± 0.03 ^bA^	0.58 ± 0.14 ^cB^	0.58 ± 0.14 ^cB^	0.52 ± 0.00 ^cB^	0.63 ± 0.05 ^bcA^
	PVA/ZMEO	1.25 ± 0.00 ^aA^	0.78 ± 0.00 ^bA^	0.75 ± 0.05 ^bA^	0.78 ± 0.07 ^bA^	0.62 ± 0.04 ^cA^	0.70 ± 0.12 ^bcA^
L*	Control	47.57 ± 0.00 ^aA^	36.52 ± 3.43 ^bC^	36.41 ± 3.30 ^bB^	35.91 ± 2.95 ^bB^	35.07 ± 0.91 ^bcB^	33.62 ± 2.11 ^cB^
	PVA	47.57 ± 0.00 ^aA^	38.54 ± 0.55 ^bB^	36.88 ± 0.52 ^bB^	36.88 ± 0.52 ^bB^	37.48 ± 0.62 ^bA^	34.39 ± 0.41 ^cB^
	PVA/ZMEO	47.57 ± 0.00 ^aA^	41.49 ± 13.26 ^bA^	39.32 ± 0.37 ^cA^	39.32 ± 0.37 ^cA^	36.32 ± 1.10 ^daB^	35.92 ± 0.68 ^dA^
a*	Control	28.02 ± 0.00 ^eA^	36.35 ± 0.06 ^aA^	33.65 ± 3.52 ^bB^	32.65 ± 6.49 ^bcC^	31.17 ± 1.30 ^cdC^	29.79 ± 3.08 ^deC^
	PVA	28.02 ± 0.00 ^cA^	36.15 ± 0.93 ^abA^	37.40 ± 2.28 ^aA^	36.90 ± 2.63 ^abB^	35.40 ± 1.16 ^abB^	34.97 ± 1.85 ^bB^
	PVA/ZMEO	28.02 ± 0.00 ^cA^	35.90 ± 0.69 ^bA^	38.87 ± 0.63 ^aA^	38.87 ± 0.63 ^aA^	37.89 ± 1.92 ^abA^	36.75 ± 0.36 ^abA^
b*	Control	28.23 ± 0.00 ^aA^	23.40 ± 1.82 ^bB^	20.65 ± 2.73 ^cB^	17.65 ± 2.02 ^dC^	16.00 ± 0.53 ^dC^	13.95 ± 1.28 ^eC^
	PVA	28.23 ± 0.00 ^aA^	24.31 ± 2.05 ^bB^	22.78 ± 1.51 ^bA^	20.78 ± 1.51 ^cB^	19.91 ± 0.96 ^cB^	16.21 ± 0.19 ^dB^
	PVA/ZMEO	28.23 ± 0.00 ^aA^	25.71 ± 1.08 ^bA^	23.61 ± 2.05 ^cA^	22.61 ± 1.34 ^cA^	23.33 ± 0.80 ^cA^	18.34 ± 0.70 ^dA^

Data shown are the mean ± standard error of three replicates. ^a–e^ Different letters indicate significant difference for the storage time for each packaging treatment. ^A–C^ Different letters indicate significant difference between packaging treatments within each storage time.

**Table 4:** Effect of active packaging using PVA fiber (PVA) and PVA fiber loaded with *Zataria Multifora* essential oil (PVA/ZMEO), and storage time on chemical traits of strawberry fruits stored at 4 °C for 15 days.

Parameter	Active Packaging Treatment	Storage Time (Day)					
		0	3	6	9	12	15
TSS (˚Brix)	Control	6.20 ± 0.42 ^aA^	5.00 ± 0.00 ^bB^	3.90 ± 0.00 ^cA^	3.52 ± 0.13 ^cB^	3.05 ± 0.24 ^dB^	3.05 ± 0.56 ^dB^
	PVA	6.10 ± 0.00 ^aA^	5.21 ± 0.18 ^bB^	3.92 ± 0.72 ^cA^	3.92 ± 0.72 ^cA^	3.35 ± 0.10 ^dA^	3.39 ± 0.03 ^dA^
	PVA/ZMEO	6.10 ± 0.00 ^aA^	5.47 ± 0.01 ^bA^	3.97 ± 0.08 ^cA^	3.97 ± 0.08 ^cA^	3.55 ± 0.14 ^dA^	3.45 ± 0.14 ^dA^
TA (%)	Control	0.72 ± 0.00 ^aA^	0.476 ± 0.01 ^bC^	0.35 ± 0.00 ^cC^	0.32 ± 0.00 ^cB^	0.26 ± 0.03 ^dC^	0.19 ± 0.02 ^eC^
	PVA	0.72 ± 0.00 ^aA^	0.526 ± 0.01 ^bB^	0.50 ± 0.03 ^bA^	0.45 ± 0.02 ^cA^	0.38 ± 0.01 ^dB^	0.27 ± 0.01 ^eB^
	PVA/ZMEO	0.72 ± 0.00 ^aA^	0.556 ± 0.00 ^bA^	0.45 ± 0.03 ^cB^	0.45 ± 0.03 ^cA^	0.40 ± 0.03 ^dA^	0.34 ± 0.03 ^eA^
Anthocyanin (mg kg^−1^ 100 g FW)	Control	273.6 ± 0.00 ^aA^	245.1 ± 14.87 ^bC^	235.1 ± 7.80 ^bcB^	226.1 ± 14.11 ^cB^	197.0 ± 9.19 ^dC^	180.0 ± 15.52 ^eC^
	PVA	273.6 ± 0.00 ^aA^	245.1 ± 0.83 ^bB^	254.1 ± 0.83 ^bA^	234.1 ± 0.83 ^cB^	207.0 ± 7.62 ^dB^	197.8 ± 15.22 ^dB^
	PVA/ZMEO	273.6 ± 0.00 ^aA^	267.2 ± 3.38 ^abA^	262.2 ± 0.14 ^abA^	257.2 ± 3.38 ^bA^	242.1 ± 14.11 ^cA^	231.0 ± 2.35 ^cA^
DPPH scavenging activity (%)	Control	35.21 ± 0.00 ^dA^	38.50 ± 4.59 ^dB^	58.50 ± 4.59 ^aC^	55.80 ± 1.97 ^abC^	53.00 ± 1.41 ^bC^	45.50 ± 1.76 ^cC^
	PVA	35.21 ± 0.00 ^eA^	40.00 ± 5.65 ^dB^	73.50 ± 1.76 ^aB^	64.00 ± 1.41 ^bB^	61.50 ± 1.76 ^bB^	49.00 ± 2.12 ^cB^
	PVA/ZMEO	35.21 ± 0.00 ^eA^	55.99 ± 3.54 ^dA^	83.50 ± 1.06 ^aA^	75.50 ± 1.76 ^bA^	70.00 ± 1.41 ^cA^	53.00 ± 2.85 ^dA^
Total phenol (mgL^−1^ GA 100g FW)	Control	1042 ± 122.5 ^deA^	1338 ± 16.44 ^aB^	1228 ± 53.03 ^bB^	1168 ± 24.74 ^bcB^	1111 ± 49.67 ^cdB^	1013 ± 15.55 ^eB^
	PVA	1042 ± 122.5 ^cA^	1430 ± 8.83 ^aA^	1288 ± 41.89 ^bB^	1221 ± 3.00 ^bB^	1239 ± 41.36 ^bB^	1097 ± 16.61 ^cA^
	PVA/ZMEO	1042 ± 122.5 ^eA^	1433 ± 10.25 ^bA^	1530 ± 9.19 ^aA^	1391 ± 123.0 ^bA^	1285 ± 38.53 ^cA^	1148 ± 26.16 ^dA^

Data shown are the mean ± standard error of three replicates. ^a–e^ Different letters indicate significant difference for the storage time for each packaging treatment. ^A–C^ Different letters indicate significant difference between packaging treatments within each storage time.

## Data Availability

Not applicable.
